# The  proportion and determinants of appropriate health seeking behavior for febrile illness among caregivers of children under-five years in Butula sub-county, Busia county, Kenya

**DOI:** 10.12688/openreseurope.18028.1

**Published:** 2024-07-10

**Authors:** Jean Louis Kananura, Betsy C.Rono, Kamija S.Phiri

**Affiliations:** 1 Environmental health and disease control, Jomo Kenyatta University of Agriculture and Technology, Nairobi, Nairobi County, Kenya; 2 environmental health and disease control, Jomo Kenyatta University of Agriculture and Technology, Nairobi, Nairobi County, Kenya; 3 School of Global and Public Health, Kamuzu University of Health Sciences, Kamuzu University of Health Sciences, Blantyre, Malawi

**Keywords:** Febrile illness, Health-seeking Behaviors, Children Under-Five Years, Individual-Level Factors, Fever.

## Abstract

**Background:**

Almost 10 million children under the age of five in Kenya, die due to fever-related diseases. In Busia, a county in Kenya, malaria accounts for 13% of all child fatalities under the age of five, a number higher than any other county. This study aimed to determine (a) proportion of appropriate health-seeking behavior and (b) determinants of health-seeking behaviors (HSBs) among their caregivers in Butula Sub-County, Busia County, as reported by the caregivers.

**Methods:**

This cross-sectional mixed-method study included 271 caregivers, 11 community health volunteers, and health facility workers in Butula Sub-County. Systematic random sampling for participants and purposive sampling for key informants were used. A questionnaire collected the data that was analysed using frequency and percentages and logistic regression.

**Results:**

70.1% of caregivers reported seeking care for a child's fever within 24 hours. Individual factors that caregivers reported to influence appropriate health-seeking HSB were unemployment (adjusted odds ratio (aOR) = 0.49, 95% CI: 0.217 – 0.593, p = 0.018), self-medication preference (aOR = 0.14, 95% CI: 0.054 – 0.363, p < 0.001), had atleast two children (aOR = 0.63, 95% CI: 0.425 – 0.937, p = 0.042), and confidence in identifying fever (aOR = 7.0, 95% CI: 2.200 – 22.439, p = 0.001). Health-system factors reported to influence HSBs were facility too far (aOR = 0.86, 95% CI: 0.526 – 0.914, p = 0.027), getting health education (aOR = 1.8, 95% CI: 1.201-4.122, p=0.015), and facility level (aOR = 4.4, 95% CI: 2.015 – 9.750, p < 0.001). Qualitative findings found health system factors related to HSB as stockouts, facility distance, and staff workload.

**Conclusions:**

Policy and practice efforts should focus on significant individual and health system determinants for HSBs among caregivers of children under five with febrile illness.

## Plain Language summary

Millions of Kenyan children below five years die from fever. Busia, a County in the Lake region of Kenya, malaria related deaths are at 13%, which is the highest in Kenya. Focusing on an area in Busia, this study looked at the percentage of caregiver who seek appropriate care and factors that might affect behaviors of caregivers to seek care for children who develop fever-related diseases. More than half of caregivers in Butula seek care for their under-five children within a day. Through surveys and interviews, caregiver and healthcare workers reported that having no job, preferring to give their children medication rather than a doctor, having more than two kids, confidence in identifying fever, hospital being too far, being educated on health, and the level of hospital where care is obtained influenced behaviors to seek care for fever. This study concludes that fever-related diseases among children under five year are above average. Efforts to improve fever among children under five years should address key factors influencing caregivers behaviors to seek care for their children.

## Introduction

Febrile illness continues to be a significant global health problem, causing approximately 75% of mortality in children under five years old ^1^. Although the World Health Organization (WHO) does not classify febrile illness as a disease, it is associated with infectious conditions such as pneumonia, malaria, and typhoid fever, which are linked with high morbidity and mortality in children under five ^1, 2^. Each year, it is estimated that about 10 million children under five years in developing countries die due to febrile illnesses ^3^. In Kenya, more than 10 million children under five years require treatment for acute febrile illnesses ^4^. In Busia County (where the study site is located), the highest rate of febrile illness and related mortality is reported in all the Kenyan counties. In 2022, the Kenya Health Information System (KHIS), for instance, reported that malaria contributed to 13% of all under-five child mortality in Busia County ^5^.

Febrile illness-related deaths are high because caregivers of children under five fail to seek timely medical care. In 2020, the Kenya Malaria Indicator Survey (KMIS) showed that the proportion of children with fever who received treatment dropped from 72% to 64% between 2015–2020 ^6^. Research shows that the behavior of seeking timely treatment significantly impacts febrile illness ^7^. In the current study, these behaviors are referred to as health-seeking behaviors (HSBs). Health-seeking behaviors are defined as any attempt by an individual (in this case a caregiver) to obtain the proper and timely treatment to address a health condition upon symptom manifestation ^8^. UNICEF and WHO, in their global action plans, recommend that caregivers obtain adequate treatment for their children to reduce the impact of febrile illness ^9^.

Many factors may determine HSBs among caregivers of children under five, including individual and health-system factors. Research on health-seeking behavior for febrile illness has revealed regional variations in health-seeking rates. According to Min *et al.* ^10^, geographic location is linked to the decision not to seek treatment for fever, with only 32.0% obtaining treatment within 24 hours. In sub-Saharan Africa, the percentage of caregivers who sought treatment for children with febrile illnesses was 67.3%, 56.8%, and 27.0% in Malawi, Tanzania, and Zambia ^11^. Odimbe and Atuhairwe, in a study conducted in the Ugandan town of Busia Municipality, revealed that factors such as illness severity and caregivers' age influenced caregivers' health-seeking behaviors for children under five years with malaria ^12^. Additionally, they identified the quality of health services and health workers' behaviors as factors influencing caregivers' HSBs to treat malaria in children under five ^12^. Liyew *et al.* ^11^ reported that knowledge, educational status, and age influenced caregivers' decision to seek treatment for febrile illness. They also reported that the availability of health facilities influenced caregivers' HSBs for children with fever ^11^.

In Kenya, poor HSBs among caregivers are demonstrated by the significant decline in the proportion of children receiving treatment for fever ^6^. Individual and health system factors may contribute to these poor HSBs. Still, there are limited comprehensive studies done in Kenya, particularly in the Western region that is prone to febrile illness, to support these claims. Therefore, this study aimed to achieve two objectives, (a) the proportion of appropriate health-seeking behavior and (b) determinants (individual and health-related factors) of health-seeking behaviors among caregivers of children under 5 years with febrile illness in Butula Sub-County, Busia County, Kenya, as reported by caregivers.

## Methods

### Study type and period

A community-based analytical cross-section study using an explanatory sequential design was employed in the study. The research assistants first collected quantitative data and later qualitative data to support the quantitative data. The study period was between May 22, 2023 and July 28, 2023.

### Study setting

The study site was Butula Sub-County, Busia County. Its one seven sub-counties in Busia County, the other being Teso North, Teso South, Nambale, Bunyala (Bundalangi), Samia (Funyula) and Matayos sub-county administrative units. Not much is published about the sub-county. Busia together with Kakamega, Bungoma, and Vihiga counties constitute the western region of Kenya. It border the Republic of Uganda to the North, Siaya to the south, Kakamega to the east, and Bungoma to the Northeast. Busia County covers an area of approximately 1,683 sq. km and is located between latitudes 00° 01' and 00° 47' North of the Equator and longitudes 33° 57' and 34° 26' East of Greenwich Meridian. Butula sub-county is prone to febrile illness-associated diseases because of being in the Victoria region basin, hence why it was purposively selected as the study area.

****Target Population.**** Caregivers of children under five years old in the study setting.

****Inclusion Criteria.**** Caregivers over 18 years, with a child under five, residing in Butula, and willing to consent.

****Exclusion Criteria.**** Caregivers who had lived in Busia County for less than two weeks before study period, were under 18 years old, and were unwilling to provide consent.

### Sampling

Butula sub-county has six wards. Three (Marachi West, Marachi Central, and Kingandole) were selected at random. The sample for the quantitative phase of the study was selected from homes with children under five using systematic random sampling. A sample size of 271 participants was used in the study. This sample was obtained using Fisher's formula with an acceptable confidence level of 95% ^13^. The prevalence or proportion used was the prevalence of fever among under-five children ^14^.


$$n_{0}=\frac{Z^{2}/pq}{e^{2}}$$

Where: n _0_ is the sample size

 Z ^2^ is the abscissa of the standard curve that cuts off an area α at the tails (1 – α), which equals the desired confidence level at 95%). 

e is the desired level of precision

p = Expected prevalence or proportion of an attribute/disease in the population from previous or pilot studies. Data on the prevalence of 17% of children below five with a fever in the two weeks before the malaria survey (KDHS, 2022)

 q is 1-p.

Z = 1.96 for a 95 % level of confidence


$$n_{0}=\frac{1.96^{2}(0.20)(1-0.20)}{0.05^{2}}$$

= 217 the sample is adjusted upwards by 10% to cushion for attrition cases

The sampling frame was determined from the sub-county register containing the number of households for caregivers under under-five. A selection interval (k) was determined by dividing the total number of households and participants. The randomly generated numbers on a computer were used to select the first household in the study each day. The 271 caregivers were allocated across the three wards at a ratio of 2:2:1.


$$\text{Interval}=\frac{\text{Households}\;\text{of}\;\text{care}\;\mathrm{givers}\;\text{of}\;\text{Under}-\text{five}\;\text{children})}{\text{Sample}\;\text{size}\;(\mathrm{calculated}\;\text{n})}$$

Healthcare workers who work in the pediatric department at Mayo Sub-county Hospital and community health volunteers providing care to children and caregivers in Butula sub-County were were purposively selected as crucial informants. Eleven key informants were found to saturate the qualitative data.

### Data collection and tools

Two research assistants collected quantitative data from 271 caregivers between May and July 2023 using a semi-structured questionnaire. The semi-structured questionnaire developed by the primary investigator was used to collect data for the quantitative phase of the study. It contained five parts that collected data on social demographic, social and economic data of caregivers, characteristics of children, the prevalence of febrile, HSBs for febrile illness, and factors influencing HSBs. Its validity and reliability were tested using 10% of the sample in the Nyalenda ward in Kisumu County, which has similar geographical characteristics and experiences high cases of febrile illness as Butula Sub-County. Cronbach's alpha was 0.86, indicating high reliability. Before distributing the questionnaire, the research assistants explained the study's purpose and objectives and obtained a signed informed consent form. An unbiased witness was present when a caregiver was incapable of reading or writing to make sure they received the correct information regarding the study. An in-person (face-to-face) approach was used to administer the questionnaire. The choice of research assistants was based on their educational background; prerequisites for inclusion were (a) a minimum degree of medical training, (b) fluency in English and Kiswahili, and (c) speaking the native language.

 A critical informant interview guide, developed by the study investigator, collected data from healthcare workers at the Mayo Sub-County Hospital. Interviews with health workers lasted 30–45 minutes and targeted institution-level factors such as turnaround time (TAT), availability of commodities, availability of human resources, distance, hospital fee, and distance. The interview session ended after reaching data saturation—FGDs with CHVs collected data on health-system-related determinants of HSBs. Tape recordings were used capture and maintain the informants exact words. A focus group discussion (FGD) guide, also developed by principal author, collected data from two FGDs, each lasting 30–60 minutes., The aim of these FGDs was to explore more into the determinants of HSB among caregivers of children under five with for febrile illness. They were conducted until data saturation.

### Data management and analysis

Quantitative data was cleaed in Excel and imported into Statistical Package for Social Scientists (SPSS) version 25 for descriptive and inferential analysis. Descriptive statistics included frequency, percent, and measures of central tendency and presented as figures and tables. A binominal logistic regression analysis was used to determine the association between individual or health-system factors and HSBs using odds ratio (OR) at 95% confidence interval and p-value less than 0.1. Qualitative data analysis began with transcribing the voice recording and transcripts. Concepts, words, phrases, and sentences were coded to identify themes through categorization. These themes formed the study results. All qualitative data analysis was manually done in Microsoft Excel 365.

## Results

In our study, 271 caregivers were enrolled between June and December 2023, and all of them responded to the questionnaire. There were no refusals, however 14 households had no one available during the survey.

### Participants characteristics

The mean age of participants was 33.4 ± 12.4 years, with most (42.1%) being between 21.3 years. 84.1% were female, and 44.6% had secondary-level education. More than half (59%) were self-employed and earned an income of less than KSH 5000 (75.3%). Most (79.3%) lived in a nuclear family, had two or fewer children (48.7%), and had one child under five years (67.5%). ( Table 1).

**Table 1.** Demographic characteristics of study participants.

	Total	Proportion (%)
**Age (years)** ≤ 20 21 – 30 31 – 40 41 – 50 51 – 60 ≥ 61	26 114 80 20 18 13	9.6 42.1 29.5 7.4 6.6 4.8
**Mean age**	*M = 33.4 year (SD = 12.4)*	
**Sex** Male Female	43 228	15.9 84.1
**Education Level** None Primary Secondary Tertiary	13 81 121 56	4.8 29.9 **44.6** 20.7
**Occupation status** Unemployed Self Formal	90 160 21	33.2 **59.0** 7.7
**Religion** Christians Others	268 03	98.9 1.1
**Residency Ward** Marachi Central Kingandole Marachi West	110 106 55	40.6 39.1 20.3
**Income (KSH)** < 5,000 5,000 – 10,000 10,001 – 20,000 20,001 – 50,000 > 50,000	204 36 13 14 4	**75.3** 13.3 4.8 5.2 1.5
**Type of Household** Grandparent Family Single-parent Nuclear Family Extended Family	12 23 215 21	4.4 8.5 79.3 7.7
**Age of the Child** ≤ 6 months 7 – 12 Months 13 – 24 Months 25 – 36 Months 37 – 48 Months 49 – 60 Months	52 63 60 49 35 12	19.2 23.2 22.1 18.1 12.9 4.4
**The mean age of the child**	*M = 21.5 months (SD =15.6)*	
**Number of Other Children** Two or less 3 – 5 Six or More	132 89 50	48.7 32.8 18.5
**The mean number of other children**	*Mean = 2.9 (SD = 2.1)*	
**Number of Children < 5 years** None One Two Three Four or More	16 183 64 6 2	5.9 67.5 23.6 2.2 0.7
**The mean number of children under 5 years**	*M = 1.4 (SD = 2.0)*	

### Prevalence of febrile illness among under-five children

**Figure 1.**
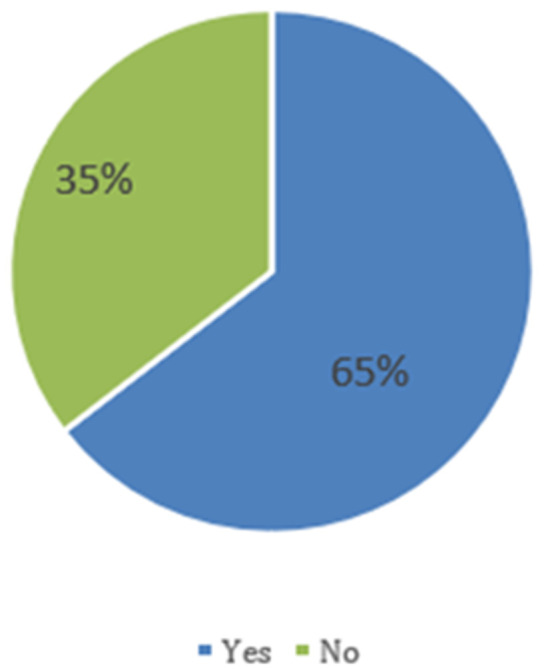
The proportion of children with fevers as reported by caregivers.

Febrile illnesses were assessed on how many children experienced fever-related illnesses in the last two weeks before the study. Figure 1 shows that 64.6% of children experienced fever episodes, and 35.4% did not.

### Proportion of appropriate Health Seeking Behavior

**Figure 2.**
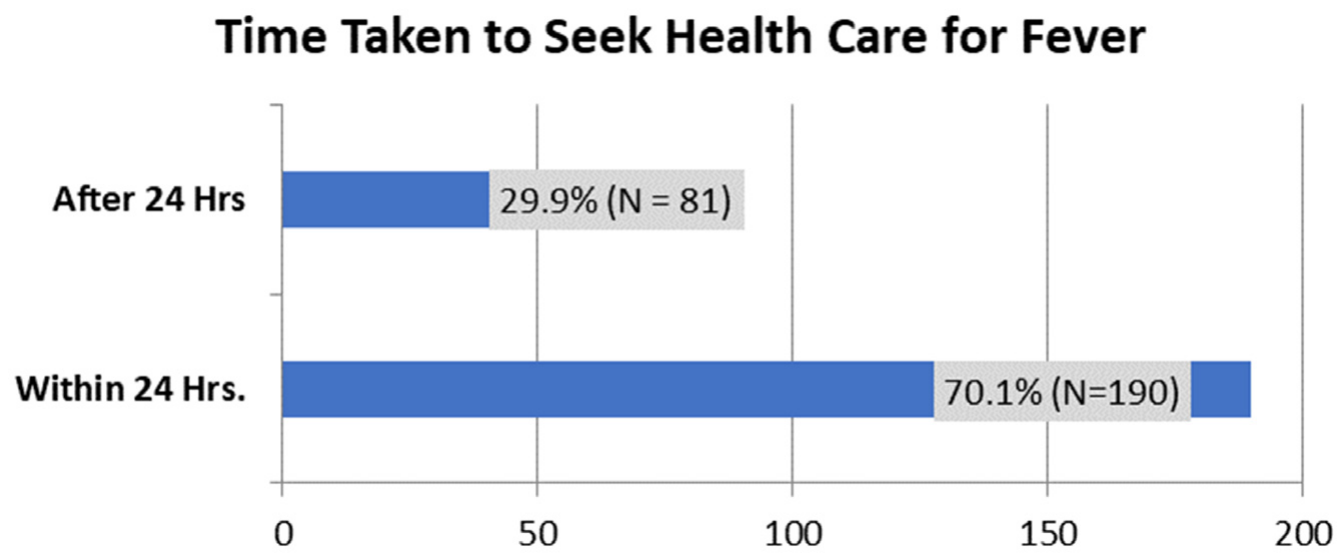
The period within which caregivers seek health care services after detecting fever in the child.

The health-seeking behavior was assessed on how long the caregiver took to seek health after detecting the child had a fever. Figure 2 shows that 70.1% of caregivers reported that they did seek healthcare within 24 hours of detecting fever in their child, while 29.9% did not.

### Determinants of Health-Seeking Behavior among caregivers

***Individual-level factors.*** The bivariate analysis of individual factors and HSB within 24 hours showed a significant association for some factors. Table 2 shows that unemployment, self-medication, personal beliefs about care, confidence in fever identification, fever severity, using a hand to identify fever, and having multiple children were significantly associated with health-seeking behavior. Being unemployed lowered the likelihood of a caregiver seeking care within 24 hours by 69% (OR = 0.41, 95% CI: 0.212 – 0.807, p = 0.011). Those who addressed fever by self-medication rather than seeking professional health had odds of caring for fever within 24 hours reduced by roughly 84% (OR = 0.16, 95% CI: 0.093 – 0.290, p < 0.001). The belief that it is good to take care of fever within 24 hours was associated with a threefold higher likelihood of seeking health within 24 hours (OR = 3.4, 95% CI: 1.441 – 8.217, p = 0.007). Those confident in identifying fever in under-five-year-old children had close to 9 times higher likelihood of having the child's fever taken care of within 24 hours (OR = 8.8, 95% CI: 3.331 – 23.049, p < 0.001). The severity of the fever increased the chances of care for fever being sought within 24 hours by two times (OR = 2.4, 95% CI: 1.401 – 4.083, p = 0.002). Using the hand to identify fever was associated with 85% chances of low HSB within the first 24 hours (OR = 0.15, 95% CI: 0.019 – 0.693, p = 0.045). Other children seemed to calm the caregiver and lower the tendency to seek care within 24 hours by 46% (OR = 0.54, 95 CI: 0.294 – 0.981).

**Table 2.** A bivariate association between individual factors and caregivers' health-seeking behavior.

Variable	Good HSB N (%)	Poor HSB N (%)	Odds Ratio (OR) (95% CI)	P -Value
**Caregiver Age** ≤ 25 Yrs. > 25 Yrs.	53 (27.9) 137 (72.1)	20 (24.7) 61 (75.3)	1.2 (0.650 – 2.142)	0.655
**Level of Education** Primary or None Secondary or Higher	63 (33.2) 127 (66.8)	31 (38.3) 50 (61.7)	0.8 (0.466 – 1.373)	0.486
**Occupation** Unemployed Employed (Self/Formal)	130 (68.4) 60 (31.6)	68 (84.0) 13 (16.0)	0.41 (0.212 – 0.807)	0.011
**Marital Status** Single/Divorced/Widowed Married	150 (78.9) 40 (21.1)	61 (75.3) 20 (24.7)	1.2 (0.761 – 1.749)	0.525
**Relationship with Child** Parent Others	156 (82.1) 34 (17.9)	67 (82.7) 14 (17.3)	0.9 (0.483 – 1.902)	1.000
**Monthly Income** ≤ Ksh. 5000 > Ksh. 5000	142 (74.7) 48 (25.3)	62 (76.5) 19 (23.5)	0.9 (0.493 – 1.667)	0.878
**How they Often Address Fever** Self-Medication CHV or Clinician	49 (25.8) 141 (74.2)	55 (67.9) 26 (32.1)	0.16 (0.093 – 0.290)	< 0.001
**Good to Take Care with 24 Hrs.** Yes No	180 (94.7) 10 (5.3)	68 (84.0) 13 (16.0)	3.44 (1.441 – 8.217)	0.007
**Can Identify Fever in Under 5** Yes No	184 (96.8) 6 (3.2)	63 (77.8) 18 (22.2)	8.8 (3.331 – 23.049)	< 0.001
**Severity Influenced HSB** Yes No	133 (70.0) 57 (30.0)	40 (49.4) 41 (50.6)	2.4 (1.401 – 4.083)	0.002
**Culture Influence HSB** Yes No	7 (3.7) 183 (96.3)	4 (4.9) 77 (95.1)	0.74 (0.209 – 2.588)	0.738
**How do you Identify fever?** Feeling by Hand Thermometer	175 (92.1) 15 (7.9)	80 (98.8) 1 (1.2)	0.15 (0.019 – 0.693)	0.045
**Has Two or More Children** Yes No	124 (65.3) 66 (34.7)	63 (77.8) 18 (22.2)	0.54 (0.294 – 0.981)	0.045
**Has other Under-five Children** **Yes** **No**	179 (94.2) 11 (5.8)	76 (93.8) 5 (6.2)	1.071 (0.360 – 3.186)	1.000

*Legend: CHV = community health volunteer, HSB-health seeking behavior, OR = odds ratio, p = p-value*

***Controlling Confounding Variables.*** Table 3 shows the adjusted odds ratio for individual factors influencing HSB within 24 hours after controlling confounders, such as unemployment, self-medication preference, having multiple children, and confidence in disease identification. Unemployment lowered health-seeking behavior rate by 51% (aOR = 0.49, 95% CI: 0.217 – 0.593, p = 0.018). Caregivers who preferred self-medication over professional care for their child's fever had 86% lower chances of seeking care within 24 hours (aOR = 0.14, 95% CI: 0.054 – 0.363, p < 0.001). Those with two or more children witness a 37% lower chance of seeking care within 24 hours than those with one or less (aOR = 0.63, 95% CI: 0.425 – 0.937, p = 0.042). Caregivers who could be confident identifying when their child had a fever were seven times more likely to seek health care services within 24 hours than those who were not confident of detecting fever (aOR = 7.0, 95% CI: 2.200 – 22.439, p = 0.001).

**Table 3.** Multivariable logistic regression on individual-level factors influencing Health-Seeking Behavior.

Variables	Crude Odds Ratio (95% CI)	P - Value	Adjusted Odds Ratio (aOR) (95% CI)	P - value
**Caregiver occupation** Unemployed Employed (Self/Formal)	0.41 (0.212 – 0.807)	0.011	0.49 (0.217 – 0.593)	0.018
**How they often address fever** Self-Medication CHV or Clinician	0.16 (0.0.93 – 0.290)	< 0.001	0.14 (0.054 – 0.363)	< 0.001
**Good to take care with 24 hours** Yes No	3.44 (1.441 – 8.217)	0.037	1.62 (0.525 – 5.017)	0.401
**Can identify fever in under 5-year-olds** Yes No	8.8 (3.331 – 23.049)	< 0.001	7.03 (2.200 – 22.439)	0.001
**Severity influenced HSB** Yes No	2.4 (1.401 – 4.083)	0.002	1.23 (0.622 – 2.418)	0.556
**How do you identify fever?** Feeling by Hand Thermometer	0.15 (0.019 – 0.693)	0.045	0.21 (0.025 – 1.801)	0.156
**Has two or more children** Yes No	0.54 (0.294 – 0.981)	0.045	0.63 (0.425 – 0.937)	0.042

*Legend: CHV = community health volunteer.*

**Figure 3.**
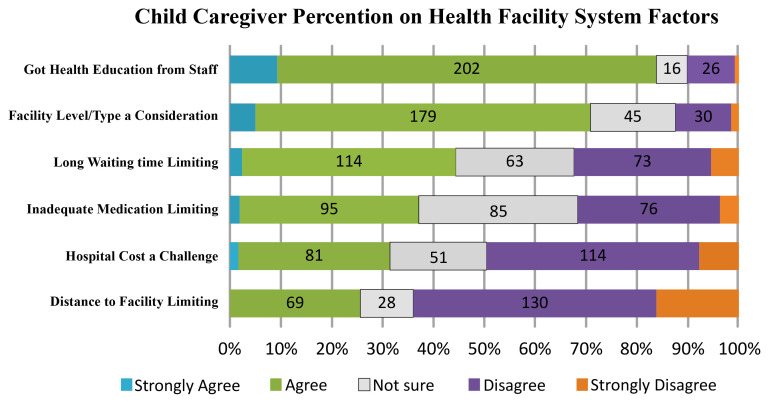
Participants’ perception on facility system factors influence on HSB.

***Health systems factors.*** Health-system factors associated with HSB based are presented in Figure 3. Based on quantitative data, most participants (84.1%, N = 228) confirmed that they had received health education from the providers on fever care while 10.0% did not. Furthermore, 71.2% (N = 193) agreed or strongly agreed to considering the facility level when deciding to seek medical attention. 44.6% (N = 121) verified that long waiting had a negative impact on the delay in seeking medical attention while 32.1% (N = 87) thought that their HSB was unaffected by extended waiting time. 37.3% (N = 86) reported that their HSB was limited by inadequate medication. 37% (N = 86) indicated that the expense of febrile treatment was a limiting factor in their HSB while 49% (N = 134) disputed this. Long distance to the facility was mentioned as a problem restricting HSB by 25.8% (N = 70) of the participants while 63.8% (N = 173) considered long distance was not an issue impacting HSB.

Multivariate analysis showed that a facility being too far was associated with lowering the HSB within 24 hours by 14% (adjusted odds ratio (aOR) = 0.86, 95% CI: 0.526-0.914, p = 0.027). Those offered health education had increased odds of having sought care within 24 hours by 82% (aOR = 1.82, 95% CI: 1.201-4.122, p = 0.015). The type/level of the facility was also a factor for consideration, increasing the access to fever care within the first 24 hours by more than four times (aOR = 4.43, 95% CI: 2.015-9.750, p < 0.001). However, cost of care (p = 0.154), long waiting time (p = 0.689), and lack of drugs in the facility (p = 0.891) had no statistical significance association ( Table 4).

**Table 4.** Bivariate and multivariate association between health seeking behavior and health system factors.

	Good HSB N (%)	Poor HSB N (%)	OR (95% CI)	p	Adjusted OR (95% CI)	p
**Facility too Far** No (reference) Yes	150 (78.9) 40 (21.1)	51 (63.0) 30 (37.0)	0.45 (0.256 – 0.802)	0.010	0.86 (0.526 – 0.914)	0.027
**Cost of Care** No (reference) Yes	135 (71.1) 55 (28.9)	50 (61.7) 31 (38.3)	0.66 (0.380 – 1.135)	0.154	-	-
**Long Waiting Time** No (reference) Yes	112 (58.9) 78 (41.1)	36 (44.4) 45 (55.6)	0.56 (0.330 – 0.942)	0.033	0.85 (0.378 – 1.902)	0.689
**Lack of Drugs in Facility** No (reference) Yes	120 (63.2) 70 (36.8)	50 (61.7) 31 (38.3)	0.94 (0.550 – 1.609)	0.891	-	-
**Staff Offered Health Education** No (reference) Yes	19 (10.0) 171 (90.0)	24 (29.6) 57 (70.4)	3.8 (1.934 – 7.423)	< 0.001	1.82 (1.201 – 4.122)	0.015
**Facility Type/Level** No (reference) Yes	44 (23.2) 146 (76.8)	34 (42.0) 47 (58.0)	2.4 (1.378 – 4.182)	0.003	4.43 (2.015 – 9.750)	< 0.001

*Legend: HSB = health seeking behavior, OR = odds ratio, p = p-value*

### Findings from critical informant interviews

Based on the interview with health workers, the following themes emerged as impacting HSB among caregivers of children under five years; medication stockouts, staffing issues, facility level, behavior of health professionals, distance from the facility, and lack of funding for outreach activities.

One health care informant said "… *caregivers preferred facilities with specialists, those that do not charge, where they can get medication, where staffs are more hospitable, those close to caregivers*," but "… *lack of funding for sensitization campaign does limit our reach to the caregivers creating a problem on identifying them*". Another one argued that "… *some prefer facilities with minimum to zero charges for services, those nearer to them, public facilities*" and further added that " *the facility has enough staff, engage the community outreaches programs, but most caregivers tend to ignore the severity of the medical issues*." The issue of drug stock out was further stressed with one interviewee saying "… *caregiver prefer to not go to hospitals since they would not be given any medication even after having been seen*".

FGDs with community health volunteers generated the following themes: caregivers' ignorance, facility accessibility, drug unavailability, poor staff motivation, and care costs. One of the FGD participants highlighted that "… *in some cases and just disturbed by the level of ignorance among caregivers not taking fever seriously in a region prone with malaria cases…."* with a fellow participant adding, "… *sometimes you feel for them, especially after knowing how bad the situation can be praying that it does not turn out to be malaria."* In terms of facility accessibility, another FGD participants noted "… *from my interaction, I think the long distance they have to cover to the facility has demotivated them to seek the health care services*." Participants agreed that the county government should do more to motivate the staff, emphasizing the im-portance of fever care and "… *authorities to recognize the significant role CHVs can have in primary care*." The social economic activity for most of the people in the region is farming, " *cost of care lowers health seeking behavior due to poverty*" and that "… *creates a problem to dispensaries and level three facilities not charging by having long waiting time and drug stock out*."

## Discussion

The aim of this study was to determine the prevalence of febrile illness among children under five years and the individual factors and health-system factors associated with health-seeking behaviors among their caregivers in Butula sub-county, Busia County. The findings have revealed that the prevalence of these illnesses is high in this community. This finding is consistent with the survey findings by KMIS who reported a prevalence of 67% ^15^ and Liyew *et al.* ^11^ who found a prevalence of 67.3% in Malawi. These is significantly higher than reported in O’Meara *et al.* ^4^ at 22.4% but slightly lower than reported by Nyaoke *et al.* ^16^ at 84%.

The study findings have revealed that individual factors influencing appropriate health-seeking behavior are unemployment, preferring self-medication over professional care, having two or more children, and confidence in identifying fever. Caregivers who reported not being unemployed were 0.49 less likely to seek appropriate healthcare. These findings support Chauhan *et al.* ^17^, who reported that occupation influences health-seeking behavior. Caregivers who could identify fever signs were seven times more likely to Have appropriate HSB. This is consistent with Zenebe *et al.* ^9^, who reported that the proportion of mothers with good knowledge of childhood illnesses was around two times more likely to seek healthcare than those with poor knowledge. Similarly, Guntur *et al.* ^7^ reported that understanding fever signs positively influences health-seeking behavior. We conclude that employment status, care preference, number of children, and caregiver confidence play a significant role in appropriate HSB among caregivers of children under 5 with febrile illness in Butula sub-county, Butula County. Interventions or initiatives to improve HSBs should consider these factors in this community and similar neighboring communities.

Additionally, the findings revealed that factors that significantly influence HSBs included staff offered health education, distance to facility, care costs, and facility type or level. There was a high probability that caregivers would engage in HSB when they received health education about febrile diseases from the facility staff. This could be explained by perceived higher confidence levels among caregivers in the nursing staff's knowledge or increased levels of trust for them to provide accurate information during the education. The healthcare facility, being level three, increased HSB twofold. Level three hospitals provide more services and have better medical equipment and other resources. According to Getahum ^18^, a lack of adequate resources is a factor determining HSB. Medication stockouts, personnel shortages, and insufficient funding for community outreach programs were associated with poor HSB ^18^. Our results are consistent with previous research. According to Muriithi *et al.* ^19^, even when a patient desired medical attention, getting there would be difficult due to the distance to the institution. Kamat ^20^ made similar observations that caregivers tend to choose nearby institutions when seeking medical attention, mostly due to the reduced cost of treatment. Ease of access to healthcare facilities is a motivator to HSB ^20^. Demand for healthcare reduces as the distance to the facility increases due to limited means of transportation and lack of transport fees ^19^. Health institutions that promoted illness education to moms were favored above those that did not ^18^. Oladigbolu *et al.* ^21^ also revealed that knowledge, medicine availability, and the high cost of care were some factors limiting HSB. We conclude that healthcare facilities being too far, staff offering education to caregivers, and facility level, particularly level 3, are key determinants of appropriate HSB among caregivers of children under 5 with febrile illness in Butula sub-county, Butula County. Therefore, action plans to improve caregivers' HSB should target these determinants to minimize the impact of these illnesses.

### Limitations

A major limitation of the study is that it was cross-sectional and therefore limited to a specific period. It does not allow for further analysis of how HSB change over time. Another limitation was that the investigators did not confirm whether caregivers’ health seeking behavior was within the indicated time and therefore relying on sincerity of the caregivers. There was potential for data collection bias.

## Conclusion

In conclusion, the prevalence of febrile illness remains high. Individual factors significantly associated with appropriate health-seeking behavior included occupation, how they address fever, ability to identify fever in under-five, and number of children per household. Based on the results of this study, staff in the health facilities should consider health education packages that target caregivers to show them the need to have a household thermometer, how to tell the child has a fever, the urgency of taking the child to the hospital, and the importance of seeking professional care as opposed to self-care in febrile illness management. Health system factors associated with HSB include facility being too far, staff workload, drug stock out, inadequate sensitization due to shortage of community outreach program, facility type, and health education. Further research should focus on longitudinal prospective cohort studies to help establish a causal-effect relationship between HSBs and individual or health-system factors, targeting Busia County in its entirety to understand the problem at the county level, possibly opening room for further investigations at the national level. 

## Ethics and consent

The Ethics Review Board at Jomo Kenyatta University of Agriculture and Technology approved the study (ref: JKU/ISERC/02316/0834) on March 9, 2023. Busia County government approved Butula Sub-County as the study site. Study participants signed the informed consent form before enrolling in the study. Privacy and confidentiality were maintained by omitting personal identifiers and storing the data in password-protected devices.

## Data Availability

Dryad: Data from: Determinants of appropriate health-seeking behavior for febrile illness among caregivers of children under 5 years in Butula sub-county, Busia County, Kenya. https://doi.org/doi:10.5061/dryad.g4f4qrfzd ^22^. The project contains the following underlying data: Jean_Louis_row_data_File---1.xlsx Jean_Louis_Coded_Data_File---1.xlsx Dryad: Data from: Determinants of appropriate health-seeking behavior for febrile illness among caregivers of children under 5 years in Butula sub-county, Busia County, Kenya. https://doi.org/doi:10.5061/dryad.g4f4qrfzd ^22^. The project contains the following extended data: Additional_file1.docx (a collection of the consent form, questionnaire for caregivers, key informant interview (KII) guide and a focus group discussion guide) README.md (summary of what the two attached Excel files contain, including abbreviations and codes) Data are available under the terms of the Creative Commons Zero "No rights reserved" data waiver (CC0 1.0 Public domain dedication).
