# Supplementary material for: The  proportion and determinants of appropriate health seeking behavior for febrile illness among caregivers of children under-five years in Butula sub-county, Busia county, Kenya
Source: Open Res Eur. 2024 Dec 12;4:143. Originally published 2024 Jul 10. [Version 2] doi: 10.12688/openreseurope.18028.2 (PMC11612550; doi:10.12688/openreseurope.18028.2)
Supplement: Peer review history

# Reviewer report: Reviewer response for version 2

**DOI:** 10.21956/openreseurope.20726.r48573

**Published:** 2024-12-27

**Authors:** Prashant Kulkarni

**Affiliations:** 1 Centre for Infectious Diseases in India CIDI, Centre for Infectious Diseases in India CIDI, Johns Hopkins school of medicine (Ringgold ID: 629744), PUNE, Maharashtra, India

1. Please clarify if it's semi-structured questionnaire or semi-structured interview schedule?

 "The respondent fills out the questionnaire on their own. A trained interviewer asks questions and records the responses. Schedules are often used to record activities or events, such as a day's schedule."

 2. Please provide details of quotes (anonymous) to bring more weight to the response.

 3. "Health-seeking behavior (HSB) is the actions people take when they perceive themselves to be ill or have a health problem".The conclusion says - the proportion of HSB was high. What does it mean? Does it mean correct HSB??

Is the study design appropriate and does the work have academic merit?

Partly

Is the work clearly and accurately presented and does it cite the current literature?

Yes

If applicable, is the statistical analysis and its interpretation appropriate?

Yes

Are all the source data underlying the results available to ensure full reproducibility?

Yes

Are the conclusions drawn adequately supported by the results?

No

Are sufficient details of methods and analysis provided to allow replication by others?

Partly

Reviewer Expertise:

Community based research; qualitative; quantitatie research; health system research

I confirm that I have read this submission and believe that I have an appropriate level of expertise to confirm that it is of an acceptable scientific standard, however I have significant reservations, as outlined above.

# Reviewer report: Reviewer response for version 1

**DOI:** 10.21956/openreseurope.19483.r46400

**Published:** 2024-12-02

**Authors:** Oluseye Ademola Okunola

**Affiliations:** 1 Medical and Health Services/ Sociology and Anthropology, Obafemi Awolowo University, Ile-Ife, Nigeria

This study explores the patterns and determinants of health-seeking behavior for febrile illnesses among caregivers of children under five in Butula Sub-County, addressing a critical gap in child health outcomes in rural Kenya. Its relevance is grounded in the high prevalence of febrile illnesses, such as malaria, which significantly contribute to morbidity and mortality in this vulnerable population.

 However, please the author should kindly check the prevalence used in calculating the sampling size in the study, p used was 0.20 instead of 0.17 according to the malaria survey (KDHS,2022).

 Also, the study design adopted should be cross-sectional, explanatory sequential mixed-methods.

 The study provides valuable insights into a specific rural setting, offering context-specific data that can inform targeted interventions. By examining both individual and systemic factors, the study captures the multifaceted nature of health-seeking behavior. Findings emphasize the need for community-level education and health system strengthening to improve timely care-seeking.

 The determinants highlighted from the study were only individual factors and health system factors other contextual factors were not succinctly elucidated.

 In conclusion , the article is well written and fills the research gap in its study context, however noting the corrections will enhance the credibility of the work.

Is the study design appropriate and does the work have academic merit?

Partly

Is the work clearly and accurately presented and does it cite the current literature?

Yes

If applicable, is the statistical analysis and its interpretation appropriate?

Yes

Are all the source data underlying the results available to ensure full reproducibility?

Yes

Are the conclusions drawn adequately supported by the results?

Yes

Are sufficient details of methods and analysis provided to allow replication by others?

Yes

Reviewer Expertise:

Health Systems and Services; Health behavior research; and Health policy

I confirm that I have read this submission and believe that I have an appropriate level of expertise to confirm that it is of an acceptable scientific standard.

# Response

**Published:** 2024-12-10

**Authors:** Jean Louis Kananura

**Affiliations:** environmental health and desease prevention, Jomo Kenyatta University of Agriculture and Technology, Nairobi, Nairobi County, Kenya

All comments have been addressed in the manuscript version 2

# Reviewer report: Reviewer response for version 1

**DOI:** 10.21956/openreseurope.19483.r46399

**Published:** 2024-11-25

**Authors:** Prashant Kulkarni

**Affiliations:** 1 Centre for Infectious Diseases in India CIDI, Centre for Infectious Diseases in India CIDI, Johns Hopkins school of medicine (Ringgold ID: 629744), PUNE, Maharashtra, India

**Reviewer’s comments**

**The  proportion and determinants of appropriate health seeking behavior for febrile illness among caregivers of children under-five years in Butula sub-county, Busia county, Kenya**

 Authors mentioned use of questionnaire method but considered interviews with community and stakeholders. Please check if it’s questionnaire or interview schedule used for the enquiry.

 Were qualitative findings congruent with survey findings?

 “… *quantitative data from 271 caregivers between May and July 2023 using a semi-structured questionnaire*”. How only quantitative data generated using semi-structured interviews?

 Participant characteristics did not bi-furcate providers and caregivers.

 Was the proportion of febrile illnesses considering all the seasons?

 In Figure-3 kindly differentiate between perception and knowledge/awareness-based data.

 The findings from healthcare workers’ interviews need to be corroborated with the survey findings.

 The discussion should be made stronger and more precise. Currently it is mixed of discussion and conclusion.

 Conclusion part includes results. Make it more concise and specific. It also includes recommendations which are not mentioned anywhere in the result section.

Is the study design appropriate and does the work have academic merit?

Partly

Is the work clearly and accurately presented and does it cite the current literature?

Yes

If applicable, is the statistical analysis and its interpretation appropriate?

Yes

Are all the source data underlying the results available to ensure full reproducibility?

Yes

Are the conclusions drawn adequately supported by the results?

No

Are sufficient details of methods and analysis provided to allow replication by others?

Partly

Reviewer Expertise:

Community based research; qualitative; quantitatie research; health system research

I confirm that I have read this submission and believe that I have an appropriate level of expertise to confirm that it is of an acceptable scientific standard, however I have significant reservations, as outlined above.

# Response

**Published:** 2024-12-10

**Authors:** Jean Louis Kananura

**Affiliations:** environmental health and desease prevention, Jomo Kenyatta University of Agriculture and Technology, Nairobi, Nairobi County, Kenya

Answer1: I mentioned questionnaire for caregivers while the key informants interview were used for the key informants from the pediatric departments, and yes the qualitative findings were congruent with survey findings(and it has been addressed in manuscript)

 Answer2: Semi-structured questionnaires were used on quantitative data collection and semi-structured interview were used on key informant (key informant interview),However it wasn’t specified in manuscript, but it now has been addressed.

 Answer3:The participants characteristics did not bi-furcate providers and caregivers because  health care providers were not main part of the study , they were care giver. Health care provider served for gathering qualitative data in order to support the study

 Answer4: No. The study was conducted in a Kenyan winter season (June, July) This The study is standard cross section study , proportion of febrile illness data  was collected by considering if they had a fever within previous two weeks

 Answer5: Depending on how questions were framed, all variables were perception based-data N.B:I have updated my conclusion and recommendation

# Reviewer report: Reviewer response for version 1

**DOI:** 10.21956/openreseurope.19483.r43554

**Published:** 2024-09-16

**Authors:** Chijioke Amara Ezenyeaku

**Affiliations:** 1 Department of Community Medicine, Nnamdi Azikiwe University, Awka, Anambra, Nigeria

**Reviewer’s report**

 Title: Appropriate and relevant

 Authors name: Not uniformly written. All the three names of the first author were written fully while the other two authors’ second names were abbreviated. The authors should choose and stick to only one format for writing all their names.

 Abstract: Background and methods were well written. The 70.1% (The proportion of caregivers that reported seeking care for a child's fever within 24 hours) under results should be written in words since sentences are not started with figures. The conclusion contained only the recommendation for improving the situation without a summary of the findings. The authors should summarize the findings of the study in one sentence before giving the recommendation.  

 Introduction: Well written and captured the background information, the statement of the problem, the justification for the study and the objectives of the study.

 Methods -

 Study setting: Line 1- ‘Its one seven sub-counties in Busia County…’ should be corrected to It is one of the seven …

 Line 4 – ‘ It border the Republic of Uganda…’ should be corrected to It borders the ….

 Exclusion criteria: Authors should note that exclusion criteria are not the opposite of inclusion criteria rather they are the characteristics found in those included in the study that will lead to their being disqualified from participating in the study. The authors should correct the exclusion criteria appropriately and also provide their explanation for excluding care-givers who had lived in Busia County for less than two weeks before the study period from the study.

 Sampling: The authors should clarify whether the three out of the six wards in Butula sub-county were selected at random or using simple random sampling technique.

 Sample size determination: p should be the proportion or prevalence obtained for the outcome variable (ie proportion of appropriate health seeking behavior for febrile illness in this instance) in a previous study rather than the prevalence of an attribute/disease (ie fever) in the population from previous studies used by authors. The authors should note this for subsequent studies.

  The authors mentioned that they used the prevalence of 17% of children below five with a fever in the two weeks before the malaria survey (KDHS, 2022) for determining the sample size. But in the formula, p was equal to 0.20 instead of 0.17. The authors should provide an explanation for this.

 Data collection and tools

 Paragraph 2 line 5 – ‘Tape recordings were used capture …’ should be corrected to Tape recordings were used to capture ….

 Data management and analysis: Line 1 – ‘Quantitative data was cleaed in Excel ..’ should be corrected to  Quantitative data was cleaned in Excel…

 Line 4 -  A binominal logistic regression analysis was used …… and p-value less than 0.1. The authors should correct this or provide an explanation for why they used p-value less than 0.1 here to denote statistical significance instead of the desired level of precision at 0.05 that they mentioned in their sample size determination.

 Results: The authors mentioned that 14 households had no one available during the survey. They should provide an explanation on what they did with those 14 households.

 Table 1: Demographic characteristics of the respondents

 The authors should explain why some of the figures were highlighted or written in bold in this table. For example – 44.6% for secondary education, 59% for self- employed, etc

 16 (5.9%) of the respondents did not have any child less than 5 years, yet they were included in this study when the inclusion criterium was care-givers over 18 years with a child under five. The authors should provide an explanation for this.

 Discussion: Line 1 – ‘The aim of this study was to determine the prevalence of febrile illness among children under five years…’ should be corrected to … to   determine the proportion of appropriate health-seeking behaviour for febrile illness among children under five years …. 

 Conclusion: Well written and drawn from the findings from the study.

 Reviewer’s impression – The article is well written and indexable. However, the authors need to address the issues raised above to make it more scientific.

Is the study design appropriate and does the work have academic merit?

Yes

Is the work clearly and accurately presented and does it cite the current literature?

Yes

If applicable, is the statistical analysis and its interpretation appropriate?

Yes

Are all the source data underlying the results available to ensure full reproducibility?

Yes

Are the conclusions drawn adequately supported by the results?

Yes

Are sufficient details of methods and analysis provided to allow replication by others?

Yes

Reviewer Expertise:

Public health, Health Systems Management

I confirm that I have read this submission and believe that I have an appropriate level of expertise to confirm that it is of an acceptable scientific standard.

# Response

**Published:** 2024-12-10

**Authors:** Jean Louis Kananura

**Affiliations:** environmental health and desease prevention, Jomo Kenyatta University of Agriculture and Technology, Nairobi, Nairobi County, Kenya

All comments are addressed in version 2(new version)
